# Local habitat conditions shaping the assemblages of vespid wasps (Hymenoptera: Vespidae) in a post-agricultural landscape of the Kampinos National Park in Poland

**DOI:** 10.1038/s41598-020-57426-8

**Published:** 2020-01-29

**Authors:** Katarzyna Szczepko, Andrzej Kruk, Bogdan Wiśniowski

**Affiliations:** 1 Department of Biodiversity Studies, Didactics and Bioeducation, Faculty of Biology and Environmental Protection, University of Łódź, Łódź, Poland; 2 Department of Ecology and Vertebrate Zoology, Faculty of Biology and Environmental Protection, University of Łódź, Łódź, Poland; 3 University of Rzeszów, Rzeszów, Poland

**Keywords:** Biodiversity, Community ecology, Conservation biology

## Abstract

The landscape of the Kampinos National Park (KPN), a UNESCO Biosphere Reserve in Poland, is a mosaic of habitats created by natural processes and human activities. However, ongoing abandonment of traditional management has led to the development of forest communities in formerly open areas. The aim of the study was to identify the local habitat conditions promoting the abundance and diversity of wasp species (Vespidae) in the post-agricultural landscape of KPN. A total of 52 samples of Vespidae caught per unit effort (CPUE) were collected across various habitats with different levels of soil humidity, bare ground and mosaicity. The highest numbers and greatest species richness of vespids were recorded in open habitats on semihydrogenic and dry soil, which provided nesting sites for hypergeic (nesting above the ground) and endogeic (ground nesting) wasps. Many solitary species that are hypergeic were significantly associated with old, abandoned wooden buildings. As vespids need resources to build and provision the nest, their communities were shaped not only by the nature of the habitat sampled but also by the mosaicity of the surrounding area. The highest abundance and species richness were recorded in samples from a heterogenous landscape, which provided a wider range of available resources in the vicinity of the nest. Our findings have significant implications for the management of park landscapes: afforestation of open habitats, both human-induced and resulting from natural succession, and the removal of old abandoned wooden buildings may limit landscape mosaicity and thus decrease hymenopteran diversity.

## Introduction

Wasps of the family Vespidae are distributed worldwide, but most of the species are known from tropical regions. With ca. 4,700 species described in the world’s fauna, the family is divided into six subfamilies^1^. In Poland, only members of three subfamilies are known: the Eumeninae, Polistinae and Vespinae^2^.

The Eumeninae wasps, also called potter wasps, belong to solitary species, and most of them are rather rarely collected. The wasps of the Polistinae and Vespinae are eusocial and include socially parasitic and workerless species which use closely related taxa as hosts^3,4^.

The social species of Vespidae are usually favoured in studies as they are very common and possess ecological and economic importance. They kill, and thus control, many pest species in order to feed their larvae. Also, they pollinate flowers that they visit to feed on their nectar^5^. Vespinae wasps pose a threat to people through their ability to sting, which can sometimes result in fatal allergic reactions to the venom^1,6^. They are also vectors of disease-causing bacteria, such as *Escherichia coli*. Wasps are sometimes responsible for losses of fruit crops, particularly plums, pears and grapes^1^.

Ecological studies concern mostly descriptions of vespid assemblages in various plant communities and ecosystems, and they lack analyses of the environmental factors determining Vespidae diversity. In addition, solitary species are often studied separately from eusocial taxa, partly because the two groups are usually treated as different families within the superfamily Vespoidea e.g.^3^.

In Poland, Vespidae inhabit both open and forested areas. The choice of a nesting site and survival of the wasps depend on (1) the availability of suitable microhabitats providing protection against unfavourable biotic (predators, parasitoids) and abiotic (moisture, rain, drought) factors; (2) food resources and (3) the availability of space or material for nest construction^5^. Eumeninae wasps make their nests in earth tunnels, hollow plant stems or old beetle borings or build mud cells in the form of small pots. The nests are provisioned with numerous small lepidopteran, beetle or symphytan larvae, which are stung and paralyzed by the females^3^. Thus provisioned, the cells are closed, and the larvae develop while feeding on the stored food. Some Eumeninae wasps show a clear preference for a particular habitat, like sandy areas, forest edges or dead wood. The Polistinae and Vespinae wasps make their paper nests both above ground (e.g. on plants, rock faces and twigs of trees and shrubs) and in subterranean locations. The developing larvae are fed by worker females, which hunt various invertebrates, mainly insects. Many species of the two subfamilies are versatile and choose various sites for nest construction^3^.

The Kampinos National Park (KNP) is a UNESCO Biosphere Reserve. Under natural conditions, open areas appear in the park as a result of either natural processes that temporarily destroy tree cover, such as fires, windfalls or outbreaks of folivorous insects, or as a consequence of human activities^7–9^. The last have been the most significant in the KNP. Its landscape has been managed consistently in a varied manner (hay-making, cattle grazing, agriculture), which provides a mosaic of habitats and ensures suitable conditions for many Vespidae species; this results in the group displaying high diversity in the KNP^10^. Such a mosaic landscape allows the females to find specific nest-building materials and food resources within the flight range from a nest site, even if they are available in different, so called partial, habitats^11^. However, ongoing abandonment of traditional management leads to development of forest communities in formerly open areas, which may threaten various hymenopteran groups, as shown in pompilid and chrysidid wasps^12,13^.

There is an urgent need to understand the requirements for preserving these ecologically important insects. Thus, the aim of the study is to identify the local habitat features to which both vespid assemblages and individual solitary and social species respond. To this end, we tested the following working hypotheses: (1) categories of sampling sites that are homogenous in terms of abiotic factor(s) do not differ in vespid abundance and richness, (2) homogenous classes of vespid samples do not differ in habitat factors at the respective sampling sites, and (3) homogenous classes of vespid samples do not differ in the number and preferences of identified indicator species.

## Material and Methods

### Study area

**Figure 1:**
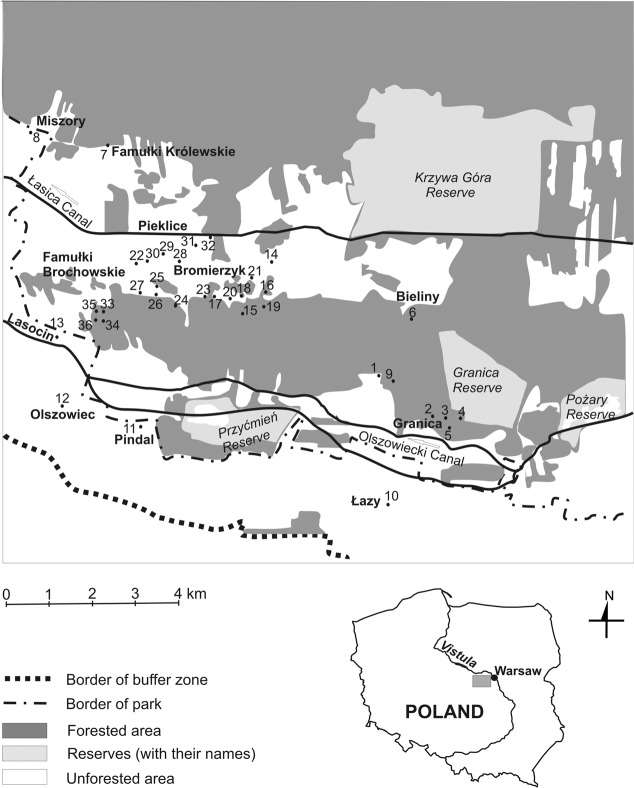
Study area with sampling sites marked with black circles. The names of the localities are in bold font.

The Kampinos National Park (KNP) (52^o^25′–52^o^15′30″N; 20^o^17′–20^o^53′E) is located on the Mazovian Lowland in Central Poland. It is one of the two national parks in Europe and one of the three in the world directly adjacent to the capital of the country. The park was created in 1959 in order to protect the unique complex of inland dunes and wetland areas, natural forest communities and rich fauna^14^. In 2000, the Park was declared a UNESCO World Biosphere Reserve “Puszcza Kampinoska” and from 2004 has also been part of the Nature 2000 network (site “Puszcza Kampinoska” PLC 140001)^15,16^. The area of KNP is 38,544 ha, including 4,636 ha of strict protection reserves (12% of the Park), and the area of its buffer zone is 37,756 ha. The Park has a belt-like structure consisting of wide belts of swampy depressions (the Łasica Canal depression and bipartite southern belt of the Olszowiecki and Zaborów Canals) separated by belts of sand dunes running parallel to the Vistula River, from east to west (Fig. 1)^14^. The swampy belts are covered by meadows, reed beds, willow shrubberies and alder-ash and alder forests. The sand dunes are among the best-preserved inland dunes in Europe. They are covered mostly by woodland (mainly pine forests); this is the dominant ecosystem in the KNP, constituting 73% of the land cover^14^. The area of the current Park has been depopulated and incorporated into the KNP by degrees since the late 1970s. The land was gradually forested or left to natural succession, with the result that its landscape is very heterogeneous. Natural habitats (forests) are interspersed with semi-natural ones (grasslands, dunes, meadows, fallow fields), and human settlements (buildings and/or farms) are either abandoned or still inhabited^14^.

The Kampinos National Park is located in the temperate zone of moderate mean latitudes. In this area, six seasons may be distinguished during the year, among which the longest is winter, with an average duration of 101 days. The growing season with temperatures exceeding 5 °C lasts for approximately 185 days a year. The mean annual air temperature is 7.7 °C, which is 1.1 °C lower than that of neighbouring areas. The KNP is characterized by a large number of days with ground frost; on average, there are 38.6 such days in the summer half of the year. The mean total annual precipitation is around 550 mm, and the mean number of days during which precipitation takes place is 124.5. The distribution of rainfall in the KNP is distinctly uneven, with lower total amounts noted in the west, and higher ones in the central and eastern parts. Westerly winds prevail^17^.

This study was carried out at 36 sites in the western part of the KNP, in Łazy (buffer zone), Bieliny, Bromierzyk, Famułki Brochowskie, Famułki Królewskie, Granica, Lasocin, Miszory, Olszowiec, Pieklice and Pindal (Fig. 1). Among the sampling sites, 13 were located on southern walls of 80- to 150-year-old wooden buildings; one on an abandoned farm with the remnants of foundations and walls, in a ruderal habitat of the *Artemisietea* class; one in fresh coniferous forest *Pino-Quercetum*; one in a degenerated bog alder forest *Ribo nigri-Alnetum*; one among old pear and apple trees in a ruderal habitat of the *Artemisietea* class; one on a sand dune, with an area of 150 m^2^ and height of 1.3 m, surrounded by mixed forest; two in psammophilous grasslands of the *Koelerio glaucae-Corynephoretea canescentis* class, one of these in a small grassland area (150 m^2^) surrounded by a woodland of oak *Quercus robur*, pine *Pinus sylvestris* and black locust *Robinia pseudoacacia*, and the other (450 m^2^) surrounded by *Cladonia* heath, *Pinus sylvestris* forest, *Betula pendula* scrub and *Robinia pseudoacacia* woodland; two were found in meadows: one in an un-mowed wet meadow of the *Calthion* alliance and another in a fresh meadow of the *Arrhenatherion* alliance mowed once a year in June and, finally, fourteen in fallow (3000–4000 m^2^) left to natural succession, the last crops being cereals or potatoes (Tables 1 and 2).

**Table 1:** Basic information on samples and sampling sites.

Sample code	Site number	Sampled habitat	Fallow age	Year of sampling	Number of other adjacent habitats	Number of species	Number of solitary species	Number of social species
01WB_00_	1	wooden building	×	2000	2	3	3	0
02WB_00_	2	wooden building	×	2000	2	5	4	1
03WB_00_	3	wooden building	×	2000	1	1	0	1
04WB_00_	4	wooden building	×	2000	2	3	2	1
05WB_00_	5	wooden building	×	2000	2	4	3	1
06WB_00_	6	wooden building	×	2000	2	4	3	1
07WB_00_	7	wooden building	×	2000	1	4	3	1
08WB_00_	8	wooden building	×	2000	1	1	1	0
09WB_01_	9	wooden building	×	2001	2	1	0	1
10WB_01_	10	wooden building	×	2001	1	3	3	0
11WB_01_	11	wooden building	×	2001	2	2	2	0
12WB_01_	12	wooden building	×	2001	2	2	1	1
13WB_01_	13	wooden building	×	2001	1	1	0	1
14AF_02_H	14	abandoned farm	×	2002	1	1	0	1
15FR_02_D	15	coniferous forest	×	2002	1	1	0	1
15FR_03_D	15	coniferous forest	×	2003	1	2	1	1
16FR_02_H	16	alder forest	×	2002	1	1	0	1
17FT_02_S	17	fruit trees	×	2002	3	8	0	8
18SD_02_D	18	sand dune	×	2002	1	3	0	3
19PG_02_D	19	psammophilous grassland	×	2002	1	4	0	4
20PG_02_D	20	psammophilous grassland	×	2002	3	4	0	4
21ME_02_H	21	meadow	×	2002	2	5	0	5
22ME_03_H	22	meadow	×	2003	1	1	0	1
22ME_04_H	22	meadow	×	2004	1	3	2	1
23FA_03_S	23	fallow	3	2003	3	10	5	5
23FA_04_S	23	fallow	4	2004	3	7	4	3
23FA_05_S	23	fallow	5	2005	3	7	3	4
24FA_03_D	24	fallow	10	2003	2	4	2	2
24FA_04_D	24	fallow	11	2004	2	5	1	4
25FA_03_S	25	fallow	4	2003	3	7	4	3
25FA_04_S	25	fallow	5	2004	3	8	3	5
25FA_05_S	25	fallow	6	2005	3	3	1	2
26FA_03_S	26	fallow	3	2003	3	5	2	3
26FA_04_S	26	fallow	4	2004	3	3	1	2
27FA_03_S	27	fallow	7	2003	3	8	3	5
27FA_04_S	27	fallow	8	2004	3	6	4	2
28FA_03_H	28	fallow	1	2003	1	2	1	1
28FA_04_H	28	fallow	2	2004	1	1	0	1
29FA_04_H	29	fallow	1	2004	1	4	2	2
29FA_05_H	29	fallow	2	2005	1	1	0	1
29FA_06_H	29	fallow	3	2006	1	4	2	2
30FA_04_H	30	fallow	2	2004	2	1	0	1
31FA_04_H	31	fallow	1	2004	1	2	1	1
31FA_05_H	31	fallow	2	2005	1	1	0	1
32FA_04_H	32	fallow	1	2004	1	3	1	2
33FA_05_D	33	fallow	10	2005	2	3	1	2
34FA_05_D	34	fallow	15	2005	2	3	0	3
34FA_06_D	34	fallow	16	2006	2	4	0	4
35FA_05_D	35	fallow	5	2005	2	3	0	3
35FA_06_D	35	fallow	6	2006	2	4	1	3
36FA_05_D	36	fallow	20	2005	2	3	0	3
36FA_06_D	36	fallow	21	2006	2	4	1	3

The code for each sample of vespid wasps consists of the site number, letters indicating the habitat (AF – abandoned farm, FA – fallow field, FR – forest, FT – fruit trees, ME – meadow, PG – psammophilous grassland, SD – sand dune, WB – wooden building), followed by two digits in subscript indicating the year the sample was collected and a letter for soil humidity level (for open areas and forests only): D – dry (autogenic), S – semihumid (semihydrogenic), H – humid (hydrogenic).

**Table 2:** Number of wasp samples assigned to SOM subclusters with respect to the type of habitats and humidity of the soil in open areas. Abbreviations of habitat names: AF – abandoned farm, FA – fallow field, FR – forest, FT – fruit trees, ME – meadow, PG – psammophilous grassland, SD – sand dune, WB – wooden building.

Subcluster	Open habitats								FR	WB	Total
	Autogenic			Semihydrogenic		Hydrogenic					
	FA	PG	SD	FA	FT	FA	ME	AF			
X_L_	1					5	1	1	3	2	13
X_W_						1				11	12
Y_D_	6	2	1		1		1				11
Y_S_	2			10		3	1				16
Total	9	2	1	10	1	9	3	1	3	13	52

### Sampling methods

A total of 52 wasp samples were collected between early April and the beginning of October from 2000 to 2006. Each sample was assigned a code consisting of (*i*) the site number (two digits), (*ii*) two letters indicating the habitat sampled (AF – abandoned farm, FA – fallow, FR – forest, FT – fruit trees, ME – meadow, PG – psammophilous grassland, SD – sand dune and WB – wooden building), (*iii*) two digits in subscript indicating the year of sample collection and (*iv*) a letter for the level of soil humidity (for open areas and forests only): D – autogenic (dry), S – semihydrogenic or H – hydrogenic (humid) (Table 1).

More than one sample was collected in successive years at some sites located in fallow areas, a meadow and a coniferous forest (Table 1). The additional samples were included in the analyses because extra effects may be revealed when a richer dataset is used.

The sampling methods were standardized. The catches of wasps were performed per unit effort (CPUE) using water-filled pan-traps; these are regarded as effective tools for collecting flower-visiting insects, including vespid wasps^18–23^. Malaise traps were not used because they have often been stolen or destroyed during previous studies and are more appropriate for ecotone habitats^24^ than the open habitats and wooden buildings in the present study. In contrast, water-filled pan-traps (plastic bowls) are simple to use, inexpensive and inconspicuous. This method is reliable, as it is independent of the diurnal activity, size or conspicuousness of insects, the weather and the experience of the researcher, all of which may considerably influence sampling results obtained from sweeping or walking a transect^22,24^.

A total of 156 plastic bowls, 20 cm in diameter, were used as traps. They were filled two-thirds full with a mixture of water (95%), glycol (5%) for preservation and a detergent to break the surface tension. At each site, three traps (two yellow and one white) were used. The two colours of traps were used because (1) yellow and white correspond to visited flowers with high reflectance^25^, and (2) various studies on the effectiveness of attracting flower-visiting Hymenoptera found that the most effective were yellow traps followed by white ones^26,27^. Depending on the type of site, they were hung on the walls of buildings, hung on trees, placed on the ground or hung on poles at a height similar to the mean height of the surrounding vegetation. In the last two cases, they were placed in the centre of the sampling site in a triangular arrangement, 10–15 m apart. Each trap was emptied every 10 days, 19 times in a given season. The aggregated 19 catches from three traps at a site were treated as one sample.

Wasps were preserved in 75% ethanol in the field and then, in the laboratory, mounted, labelled and deposited at the Department of Biodiversity Studies, Didactics and Bioeducation, University of Łódź. Their identification was based on^28–30^. The nomenclature of the insects follows Fauna Europaea^31^.

### Categories of species and habitat features

The species were classified into one of the following three groups according to their environmental preferences: a) eurytopic, associated with open areas; b) thermophilous, associated with open areas; and c) associated with woodland areas and forest edges. They were also classified into the following three guilds based on their nesting behaviour: *i*) endogeic (nesting in the ground); *ii*) endogeic/hypergeic (nesting in or above the ground) and *iii*) hypergeic (nesting above the ground). Information on the preferred habitats and nesting habits of each species was gained from Archer^1^, Blüthgen^4^, Schmidt and Schmid-Egger^32^, Schedl^33^, Dylewska and Wiśniowski^34^, Esser *et al*.^35^, Sawoniewicz and Wiśniowski^36^, Witt^37^, Macek *et al*.^38^.

The soil et each site was classified into one of the three following types: hydrogenic (humid), i.e. moorish or muckous soil; semihydrogenic, i.e. brown soils or black earth; autogenic (dry), i.e. podzolized soil or podzol. The soil type was determined with ArcGIS ver. 9.3.1 software by superimposing geographic GPS (Garmin GPSMap, 60Cx) coordinates of sampling sites on GIS soil maps^39^. This analysis was supplemented with descriptive information on the soil types from Konecka-Betley^40^ and Piórkowski *et al*.^41^. The percentage of bare ground was categorised as G1-G4: G1 (0–25% of exposed surface), G2 (26–50%), G3 (51–75%), and G4 (76–100%). The mosaicity of the vicinity of each site was expressed as the number of habitats adjoining the habitat sampled; the observed values ranged from 1 to 3 (Table 1).

### Pattern recognition

The patterns in the abundance of vespid wasps were determined using a Kohonen artificial neural network (ANN)^42,43^. ANNs are simple structural and functional models of a brain. They are built of processing units called neurons or nodes. They do not require *a priori* specification of the model underlying the studied phenomenon because they learn features from the dataset themselves^44,45^. ANNs easily deal with variables that are distributed in a skewed fashion and related in a complex way. This is especially useful in analyses of wasp counts, which often do not exhibit a normal distribution, because of the many zeroes among the datasets, and cannot be normalised with any transformation^46^.

It is worth mentioning that there are a few studies employing SOMs on hymenopterans like ants^47,48^, polistinae wasps^49^ or spider and cuckoo wasps^12,13^. However, the present study is the first to use Kohonen ANNs to examine the patterns in vespid assemblages.

**Figure 2:**
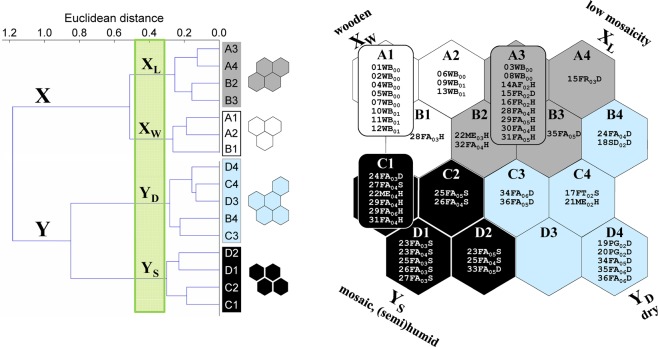
The 16 SOM output neurons arranged in a two-dimensional grid (4 × 4) with 52 real samples of vespid wasps assigned. Clusters (X and Y) and subclusters (X_L_ and X_W_, and Y_D_ and Y_S_; shaded) of neurons (and respective virtual samples) were determined by hierarchical cluster analysis. The adjectives next to subcluster symbols refer to habitat characteristics (compare with Fig. 3). The code for each vespid sample consists of the site number and two letters for the habitat (AF – abandoned farm, FA – fallow, FT – fruit trees, FR – forest, ME – meadow, PG – psammophilous grassland, SD – sand dune, WB – wooden building); each code ends with two digits in subscript indicating the year in which the sample was collected and a letter for the level of soil humidity (for open areas and forests only): D – autogenic (dry), S – semihydrogenic, H – hydrogenic (humid).

Kohonen ANNs are also referred to as self-organizing maps (SOMs). They are used for recognizing patterns in datasets. Kohonen ANNs are built of two (input and output) layers of neurons. The number of input neurons is equal to the number of variables: in this study, the log transformed abundances of 24 taxa. The output neurons were arranged on a two-dimensional grid measuring 4 × 4 neurons, which was selected from among other tested arrangements (Fig. 2). During network training, the dataset (24 taxa × 52 samples) was repeatedly presented to the input neurons. Each input neuron was connected and repeatedly transmitted information to each output neuron. The input neurons had no further significance for pattern recognition^50^. On the basis of strengthened or weakened intensity (weight) of signals obtained from the input neurons, a virtual sample of vespid wasps (SVW), understood as a set of abundances of the 24 species, was created in each output neuron. The dissimilarity of the virtual SVWs was reflected by their position on the SOM: virtual SVWs in distant regions of the SOM differed considerably, whereas virtual SVWs in neighbouring neurons were similar. Additionally, virtual SVWs, and hence their respective output neurons, were clustered by hierarchical cluster analysis (Ward linkage method, Euclidean distance)^51^. Each real SVW was finally assigned to the best matching virtual SVW and the corresponding output neuron. Consequently, dissimilar real SVWs were located in distant neurons, whereas similar real SVWs were located in the same neuron or in neighbouring ones.

The network training and clustering of virtual SVWs was performed using the SOM Toolbox^52^, developed in the Laboratory of Information and Computer Science at the Helsinki University of Technology. The SOM Toolbox allows the relative abundance of each species in virtual SVWs in the output neurons to be visualised in the form of greyness intensity, resulting in the creation of a greyness pattern for each species on the SOM. Species with similar greyness patterns usually have similar habitat preferences.

The SOM Toolbox also enables visualisation of the environmental variables in the output neurons as greyness intensity. It should be emphasized, however, that environmental variables were not presented to the Kohonen ANN and therefore did not directly influence the classification. The greyness intensity in each output neuron reflected the mean rank of a given abiotic factor relating to the real SVWs assigned to it.

### Indicator species analysis

Because the SOM Toolbox does not verify the above associations statistically, indicator species analysis (ISA), based on the indicator value (IndVal) by Dufrêne and Legendre^53^ was applied (based on the untransformed vespid abundance data). The IndVal allows the indicator vespid species to be identified for each SOM region, i.e. species significantly (*p* ≤ 0.05) associated with each SOM (sub)cluster of real SVWs. IndVal (0–100%) of the species *i* for all real SVWs of (sub)cluster *j* is a product of (*A*_*ij*_) mean abundance of species *i* in real SVWs assigned to (sub)cluster *j* over the sum of its average abundances in all (sub)clusters (%), (*F*_*ij*_) the frequency of species *i* (%) in real SVWs assigned to (sub)cluster *j* and the constant 100 in order to obtain percentages as follows:

$$\begin{array}{ccc}{{\rm{A}}}_{ij} & = & {{\rm{a}}{\rm{b}}{\rm{u}}{\rm{n}}{\rm{d}}{\rm{a}}{\rm{n}}{\rm{c}}{\rm{e}}}_{ij}/{{\rm{a}}{\rm{b}}{\rm{u}}{\rm{n}}{\rm{d}}{\rm{a}}{\rm{n}}{\rm{c}}{\rm{e}}}_{i.}\\ {{\rm{F}}}_{ij} & = & N\,{{\rm{s}}{\rm{a}}{\rm{m}}{\rm{p}}{\rm{l}}{\rm{e}}{\rm{s}}}_{ij}/{\rm{N}}\,{{\rm{s}}{\rm{a}}{\rm{m}}{\rm{p}}{\rm{l}}{\rm{e}}{\rm{s}}}_{.j}\\ {{\rm{I}}{\rm{n}}{\rm{d}}{\rm{V}}{\rm{a}}{\rm{l}}}_{ij} & = & {{\rm{A}}}_{ij}\times {{\rm{F}}}_{ij}\times 100\end{array}$$

IndVal had a maximum (100%) when all real SVWs with a given species were in a single (sub)cluster of neurons, and when the species was recorded in all real SVWs assigned to that subcluster^53^. The IndVals were calculated, and their significance levels were determined with a Monte Carlo randomisation test using PC-ORD statistical software^54^.

Hence, the ISA and SOM species planes complement each other by expressing the importance of each SOM region to a species, numerically in the former and as greyness patterns in the latter, in terms of the environmental conditions corresponding to the assigned samples or sites. Both approaches allow identification of the (sub)clusters of neurons in which a given species is most frequent and/or abundant and hence, the abiotic conditions that it prefers.

### Comparison between groups

Using the Kruskal-Wallis test and the *post hoc* Dunn test^46,55^ the abundances and species richness of wasps were compared according to the following four dimensions: SOM subclusters, categories (H1-H3) of soil humidity, categories (G1-G4) of exposed surface and whether the site was located in a habitat neighbouring one, two or three others (respectively, categories M1-M3).

## Results

A total of 3563 individuals of vespids were captured. They represented 24 species, including 15 species of solitary wasps (160 specimens) and nine species of social wasps (3403 specimens) (Table 3). The highest number of species was recorded on fallow land (14; seven on hydrogenic soil, eight on autogenic soil, and 13 on semihydrogenic soil) and wooden buildings (13), with the lowest numbers observed in a forest and on a sand dune (three at each) and on an abandoned farm (one). Five species were captured on psammophilous grasslands, seven on meadows and eight on fruit trees.

**Table 3:** Relative abundance (A), relative frequency (F) and IndVals (I) (all in %) and total observed abundance (TA, expressed as the number of specimens) of Vespidae wasps divided into two groups: species recorded in ≥3 samples (α) and remaining species (β). Significant (*p* ≤ 0.05) IndVals are underlined (exact significance levels are presented in Fig. 5). Information on habitat preferences is marked by a letter preceding the species name (a – eurytopic, associated with open areas; b – thermophilous, associated with open areas; c – associated with woodland areas and forest edges); SL – solitary species, SC – social species.

Subcluster		Species α																Species β							
		c. *Symmorphus bifasciatus* ^SL^	c. *Euodynerus notatus* ^SL^	a. *Ancistrocerus nigricornis* ^SL^	a. *Ancistrocerus claripennis* ^SL^	c. *Symmorphus murarius* ^SL^	a. *Ancistrocerus trifasciatus* ^SL^	a. *Polistes dominula* ^SC^	a. *Vespula germanica* ^SC^	a. *Vespula vulgaris* ^SC^	c. *Vespula rufa* ^SC^	c. *Vespa crabro* ^SC^	a. *Polistes nimpha* ^SC^	a. *Eumenes coronatus* ^SL^	b. *Eumenes coarctatus* ^SL^	b. *Eumenes pedunculatus* ^SL^	b. *Allodynerus delphinalis* ^SL^	a. *Ancistrocerus antilope* ^SL^	a. *Ancistrocerus gazella* ^SL^	c. *Symmorphus allobrogus* ^SL^	c. *Symmorphus connexus* ^SL^	c. *Dolichovespula media* ^SC^	c. *Dolichovespula saxonica* ^SC^	c. *Dolichovespula adulterina* ^SC^	b. *Microdynerus parvulus* ^SL^
	TA	9	10	3	5	5	6	38	78	76	3	12	3190	5	67	32	8	2	1	2	1	1	4	1	4
X_L_	A	21	0	0	0	0	0	0	1	7	0	0	8	0	0	0	29	0	0	0	0	0	0	0	0
	F	15	0	0	0	0	0	0	8	23	0	0	85	0	0	0	8	0	0	0	0	0	0	0	0
	I	3	0	0	0	0	0	0	0	2	0	0	7	0	0	0	2	0	0	0	0	0	0	0	0
X_W_	A	79	92	73	100	100	57	59	0	0	0	0	0	25	0	4	0	100	100	100	100	0	0	0	0
	F	17	17	17	42	25	25	67	0	0	0	0	8	8	0	8	0	17	8	17	8	0	0	0	0
	I	13	15	12	**42**	**25**	14	**40**	0	0	0	0	0	2	0	0	0	17	8	17	8	0	0	0	0
Y_D_	A	0	0	0	0	0	0	15	50	79	100	67	20	0	6	0	0	0	0	0	0	100	100	100	0
	F	0	0	0	0	0	0	27	91	91	27	36	100	0	27	0	0	0	0	0	0	9	9	9	0
	I	0	0	0	0	0	0	4	**46**	**72**	**27**	**24**	20	0	2	0	0	0	0	0	0	9	9	9	0
Y_S_	A	0	8	27	0	0	43	25	49	14	0	33	71	75	94	96	71	0	0	0	0	0	0	0	100
	F	0	6	6	0	0	13	50	63	38	0	25	100	25	75	81	31	0	0	0	0	0	0	0	13
	I	0	0	2	0	0	5	13	30	5	0	8	**71**	**19**	**70**	**78**	**22**	0	0	0	0	0	0	0	13

Similar results were obtained regarding the variability of wasp abundance, i.e. the highest values were recorded on fallow land (mean 116.8 specimens per sample, ranging from 46.5 on hydrogenic soil, to 78.1 on autogenic soil, and to 214.8 on semihydrogenic soil), on fruit trees and meadows (39 specimens on both) and in psammophilous grasslands (27.5), while the lowest values were observed on the sand dune (12 specimens) and on the abandoned farm and in forests (1 and 2.6 specimens, respectively). The only exception was wooden buildings, with high species richness (13, including 11 solitary species) but low abundance (4.7 specimens).

As the dominant species *Polistes nimpha* (Christ, 1791) comprised 89.5% of all collected specimens, the next most abundant species were much rarer: *Vespula germanica* (Fabricius, 1793) – 2.19%, *Vespula vulgaris* (Linnaeus, 1758) – 2.13%, and *Eumenes coarctatus* (Linnaeus, 1758) – 1.88%, *Polistes dominula* (Christ, 1791) – 1.07% and *Eumenes pedunculatus* (Panzer, 1799) – 0.90%.

In the output layer of the SOM, two clusters of neurons X and Y (X: neurons A1-A4 and B1-B3; Y: B4, C1-D4) were identified by hierarchical cluster analysis (Fig. 2). Each of these clusters included two subclusters, which were ordered according to the gradients observed in total abundance and number of vespid species: X_L_ (neurons A3, A4, B2, B3) and X_W_ (neurons A1, A2, B1) in cluster X, and Y_D_ (neurons B4, C3, C4, D3, D4) and Y_S_ (neurons C1, C2, D1, D2) in cluster Y. The subscript letters in the subcluster symbols refer to habitat characteristics (L – low mosaicity, W – wooden, i.e. buildings, D – dry, S – (semi)humid and mosaic). The numbers of wasp samples assigned to neurons in the subsequent subclusters were 13, 12, 11 and 16 (Fig. 2, Table 2).

The SOM subclusters differed considerably regarding the spatial origin of the samples. Subcluster X_L_ included all the samples from forests, samples from open habitats on mostly hydrogenic soil and two samples from wooden buildings, while all the samples in subcluster X_W_ were collected on buildings, apart from one sample (28FA_03_H), which was collected from fallow ground on hydrogenic soil (Table 2). Subcluster Y_D_ was composed entirely of samples from open habitats on autogenic (dry) soil, with two exceptions, and Y_S_ comprised samples mainly from fallows, most of which were located on semihydrogenic and hydrogenic soil (Fig. 2, Table 2).

**Figure 3:**
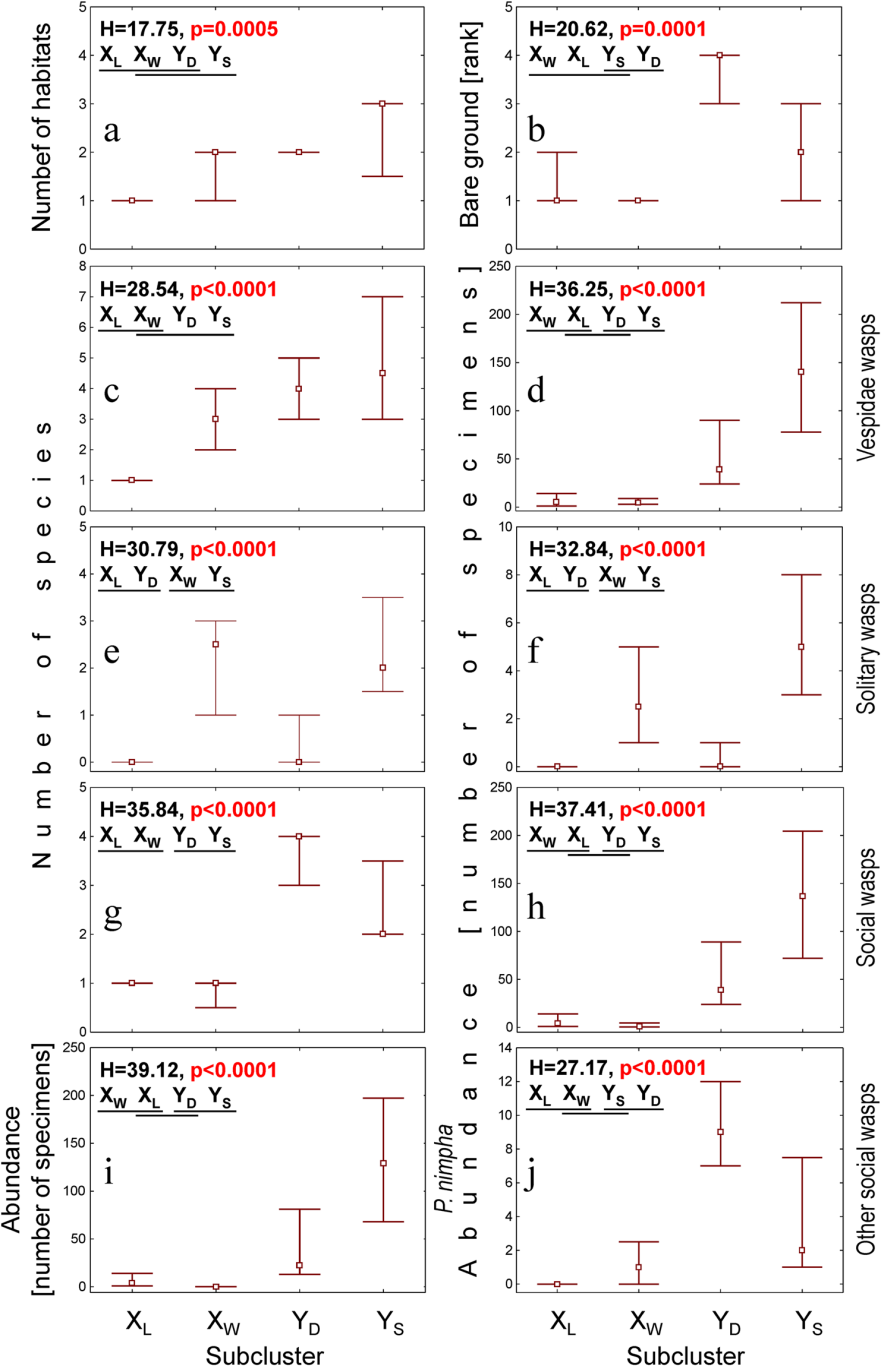
The landscape mosaicity (expressed as the number of habitats adjoining the habitat sampled) (**a**), the availability of bare ground (**b**), the species richness and abundance of Vespidae (**c**,**d** – all; **e**,**f** – solitary; **g**,**h** – social Vespidae; **i** – the abundance of dominant *Polistes nimpha*, **j** – the abundance of social Vespidae without *P. nimpha*) in SOM subclusters X_L_-Y_S_. Point – median, whiskers – inter-quartile range, H – the Kruskal-Wallis test statistic (df = 3, N_XL_ = 13, N_XW_ = 12, N_YD_ = 11, N_YS_ = 16), which was used for inter-subcluster comparisons. The subclusters underlined by the same line were not significantly different in *post hoc* comparisons.

In the subsequent subclusters (X_L_, X_W_, Y_D_, and Y_S_) an upward trend was observed in the number of habitats adjacent to the sampled one, which was used as a measure of landscape mosaicity (Fig. 3a); in addition, significant differences were observed between X_L_ and Y_S_. A similar trend was observed in the species richness of vespids (Fig. 3c), and the abundance of both all wasps and social wasps (both groups being considerably influenced by the abundance of the dominant *Polistes nimpha*) (Fig. 3d,h,i). Significant differences were also observed in the availability of bare ground (exposed surface) between (1) X_W_, X_L_ (lowest medians) and (2) Y_D_ (highest median) (Fig. 3b). A similar pattern was observed for the median numbers of species of social wasps (Fig. 3g), while to a certain extent, the opposite was observed for the species richness and abundance of solitary wasps (Fig. 3e,f).

**Figure 4:**
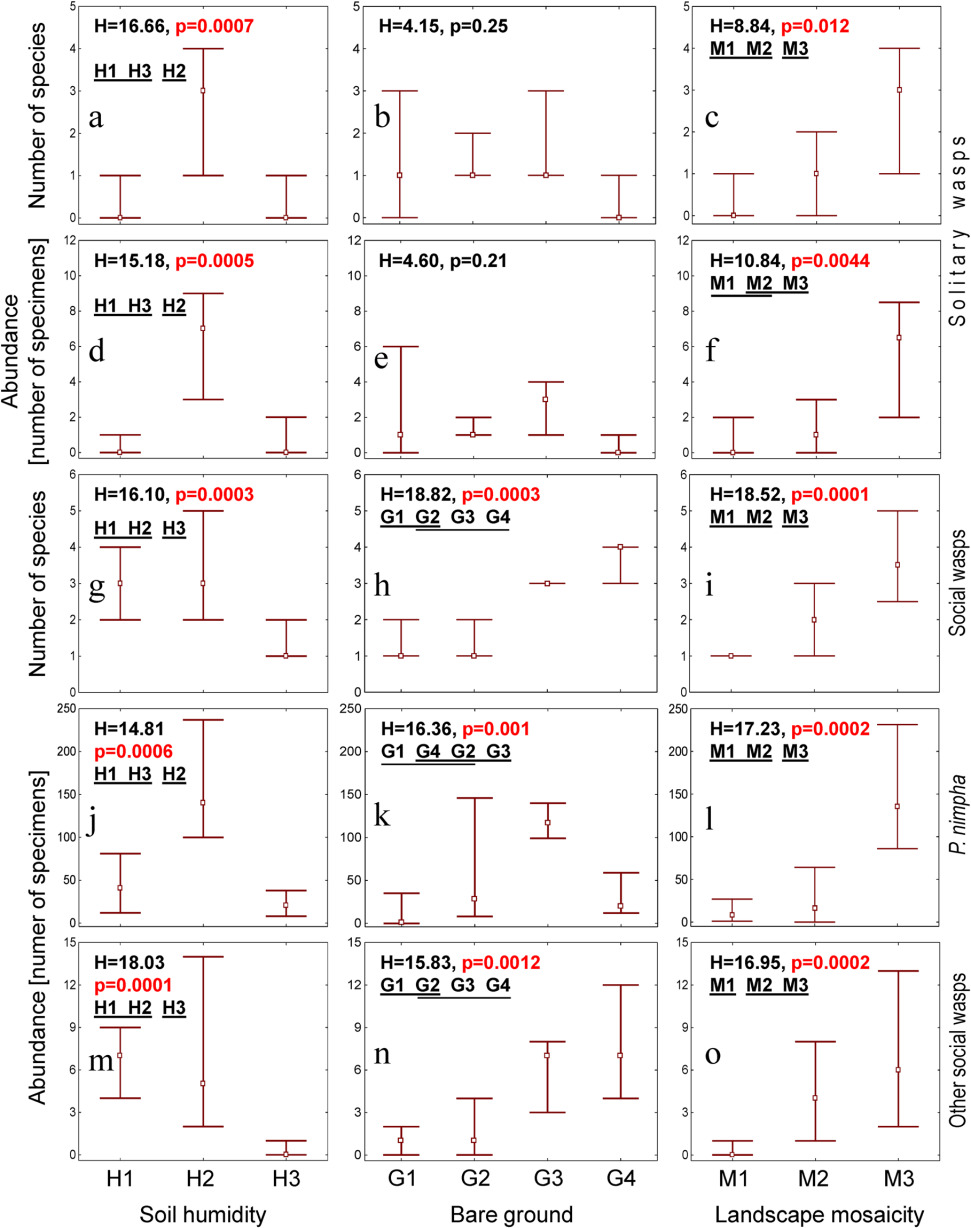
The species richness and abundance of the Vespidae species in relation to the soil humidity (left column), availability of bare ground (middle column) and mosaicity of the landscape (right column): the number of species of solitary wasps (**a**–**c**), their abundance (**d**–**f**), the number of species of social wasps (**g**–**i**), the abundance of the dominant *Polistes nimpha* (**j**–**l**) and the abundance of social wasps without *P. nimpha* (**m**–**o**). Soil humidity: H1: dry (autogenic), H2: semihumid (semihydrogenic), H3 – humid (hydrogenic). Bare ground: G1: ≤25%, G2: 26–50%, G3: 51–75%, G4: >75%. The number of habitats neighbouring the habitat sampled: M1: 1, M2: 2 and M3: 3. H – the Kruskal-Wallis test statistic (N_G1_ = 27, N_G2_ = 9, N_G3_ = 9, N_G4_ = 7; N_M1_ = 21, N_M2_ = 19, N_M3_ = 12; N_H1_ = 14, N_H2_ = 11, N_H3_ = 14). The remaining explanations as in Fig. 3.

We managed to identify individual abiotic factors that influenced vespids. One of them was soil humidity, which was analysed for open habitats only. Semihumid soils were favourable to solitary wasps and social *P. nimpha*, while both dry and semihumid soils promoted high diversity and abundance of the remaining social wasps (Fig. 4a,d,g,j,m). Habitats on humid soils were avoided by both groups. A higher availability of bare ground positively influenced only social species (Fig. 4h), including the dominant *Polistes nimpha*, which increased only in the first three categories of availability of bare ground (Fig. 4k). This factor was found to have very little influence on the species richness or abundance of solitary wasps (Fig. 4b,e). Increased landscape mosaicity positively influenced both solitary and social wasps (Fig. 4c,f,i,l,o).

**Figure 5:**
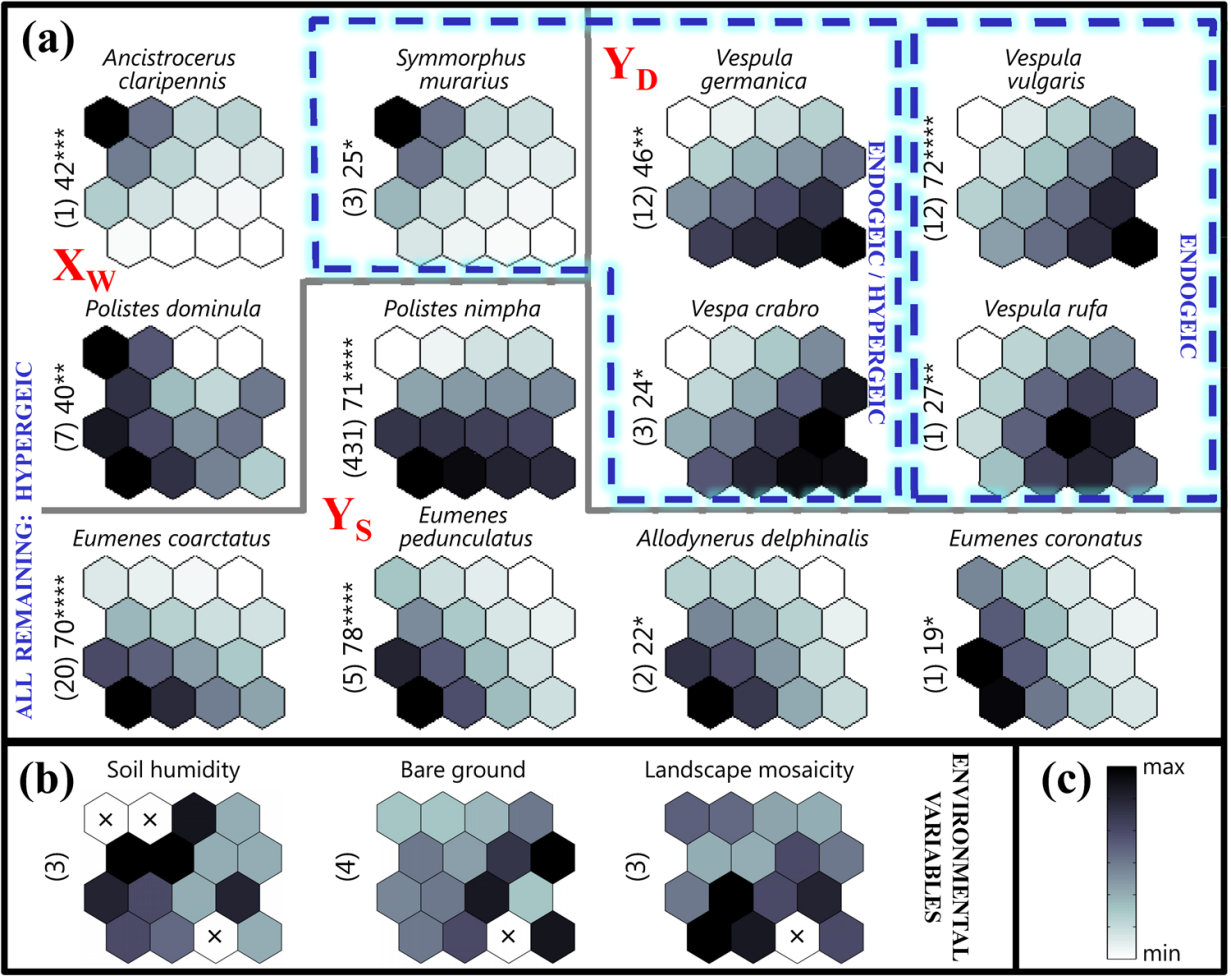
The intensity of biological and environmental variables in the SOM regions (subclusters of output neurons; see Fig. 2). (**a**) The associations (*p* ≤ 0.05) of 12 indicator vespid species with the SOM regions (greyness based on virtual samples of vespid wasps). There were no such species for subcluster X_L_. The maximum indicator value (IndVal; based on real samples of vespid wasps) recorded for a given species and the respective significance level (*p* ≤ 0.05; **p ≤ 0.01; ***p ≤ 0.001; ****p ≤ 0.0001)) are presented on the left side of each species plane outside parentheses. Species with similar patterns over the SOM occurred in similar environmental conditions. (**b**) Selected environmental variables. × – empty neuron (i.e. without any assigned sample; see Fig. 2) or values not determined. The remaining explanations as in Fig. 4. (**c**) The scale. Shading (based on virtual samples in (**a**) and initial data in (**b**)) was scaled independently for each variable. The real values varied from zero to the maximum value presented on the left side of each plane in parentheses.

Among the 16 species that were recorded non-sporadically, 75% were found to be indicators, i.e. they exhibited significant maximum IndVals. No indicator or specific species was found for X_L_ (Fig. 5, Table 3). The number of indicator species increased in the subsequent subclusters from 0 for X_L_ to 3 for X_W_, 4 for Y_D_ and 5 for Y_S_ (Fig. 5, Table 3), which resembles the trend observed above for landscape mosaicity (Figs. 3a,c,d,h,i, and 5). Most of the species with significant maximum IndVals (in group α), or which were caught sporadically and were present in samples assigned to only one subcluster (group β), were solitary in the case of X_W_ and Y_S_ but social in the case of Y_D_ (Table 3); these findings are in line with the patterns observed in the species richness of both social and solitary wasps and in the abundance of the social wasps, excluding *P. nimpha*, and the solitary wasps (Fig. 3e,f,g,j).

While most indicator species of cluster X (X_W_) tended to be eurytopic species and those preferring woodland areas and forest edges (groups a and c), two solitary and one social, the indicator species of cluster Y (Y_D_, Y_S_) included species from all three groups (a–c), all of which were social in Y_D_, and mostly solitary in Y_S_ (Table 3).

As far as the nesting preferences are concerned the endogeic species (guild *i*) were significantly associated only with subcluster Y_D_, the species nesting in or above the ground (guild *ii*) were associated with subclusters X_W_ and Y_D_, and the hypergeic species (guild *iii*) were associated with subclusters X_W_ and Y_S_ (Fig. 5, Tables 3 and 4).

**Table 4:** Species of wasps recorded in ≥3 samples (compare with Table 3) classified according to their nesting behaviour: *i*) nesting in the ground, *ii*) nesting in or above the ground and *iii*) nesting above the ground. The subcluster with the highest IndVal is given (and underlined if p < 0.05) for each species.

Group	Species	Subcluster
*i*	*Vespula vulgaris*	Y_D_
	*Vespula rufa*	Y_D_
*ii*	*Vespula germanica*	Y_D_
	*Vespa crabro*	Y_D_
	*Symmorphus murarius*	X_W_
	*Euodynerus notatus*	X_W_
*iii*	*Symmorphus bifasciatus*	X_W_
	*Ancistrocerus nigricornis*	X_W_
	*Ancistrocerus trifasciatus*	X_W_
	*Ancistrocerus claripennis*	X_W_
	*Polistes dominula*	X_W_
	*Polistes nimpha*	Y_S_
	*Eumenes coarctatus*	Y_S_
	*Eumenes coronatus*	Y_S_
	*Eumenes pedunculatus*	Y_S_
	*Allodynerus delphinalis*	Y_S_

## Discussion

The number of species recorded during this study represents nearly 40% of all Vespidae species recorded so far in Poland, these representing 64% of social wasp species and 31% of solitary wasps^2,56^. This proportion is similar to that recorded for other Aculeata taxa in the Kampinos National Park (*ca*. 50% pompilids and 46% chrysidids)^12,13^. The number of vespid species known presently in the KNP is 35^10^, which is a higher value than that found in most other national parks (NP) in Poland, e.g. 21 species in the Pieniny NP, 24 in each of the Białowieża and Wielkopolski NPs, 42 in the Wigry NP^57,58^ and 44 in the Ojców NP (Wiśniowski *unpublished data*). This richness of vespid species results from the presence of diversified open areas mixed with various types of forests and reflects the status of the KPN, together with its buffer zone, as one of the most important faunal refugia in the Polish lowlands^14^.

Of the six dominant vespid species of KNP, four are eusocial species forming large colonies: a strategy which enhances their potential to reach high population abundances. Because they have such varied habitat requirements, the high landscape mosaicity of the study area may favour the high abundances of these species. Two of them, the eurythopic *Vespula germanica* and *V. vulgaris*, are dominants or subdominants in many other studies conducted in various habitats: from natural or anthropogenic open ones, which are slightly more preferred by *V. germanica*, to forests with little predominance of *V. vulgaris*^20,59–66^.

Studies on the Kampinos NP have identified two other dominant social species which create smaller colonies: *Polistes dominula* and *P. nimpha*. Species of the genus *Polistes* are also referred to as eurythopic but associated with open habitats and are regarded as being more thermophilous than *Vespula vulgaris* and *V. germanica*^28,34,67,68^. Our present findings indicate that *Polistes nimpha* exhibited very high dominance, reaching almost 90% of all vespids in KPN; this was unexpected because many other studies have found it to be represented by few specimens^20,65,69^ or even absent^19,61–63,70–72^. Even in the Kampinos NP, *Polistes nimpha* was not noted in previous research conducted during 1988–1992. The relation of abundances of these two species may serve as a bioindicator of the state of a given ecosystem because *Polistes dominula* can be described as synanthropic, whereas *P. nimpha* prefers more natural habitats. The current dominance of *Polistes nimpha* indicates the renaturalisation of the study area after it was released from human impact.

*Polistes dominula* was considered critically endangered (CR) by Skibińska^73,74^, who treated it as a synonym of *P. gallicus* [syn. *P. gallicus* Linnaeus, 1767]. In view of the above, and the increasing knowledge of the distribution of the species in Poland, Oleksa and Wiśniowski^67^ proposed VU (vulnerable) or LR (lower risk) categories for *Polistes dominula*. The authors also emphasised the need for better recognition of ecological preferences for this wasp under the conditions found in Poland. In addition, two solitary vespid species recorded in this study, *Allodynerus delphinalis* (Giraud, 1866) and *Euodynerus notatus* (Jurine, 1807), are included on the Red Data List of threatened species^75^. Three other species are believed to be rare in Poland: the solitary *Eumenes coarctatus* (another dominant in this study), *E. coronatus* (Panzer, 1799) and the social parasite *Dolichovespula adulterina* (du Buysson, 1905)^2^.

The species richness and abundance of vespids were the lowest in subcluster X_L_, i.e. samples from fallow fields, an abandoned farm and a meadow, often on humid soil, and from forests (Figs. 2 and 3). Earlier studies have also shown that wasps avoid wet habitats such as wetlands^72^ or open habitats on hydrogenic soils^12,13^. Wet meadows are unattractive for aculeates and are used more for foraging than for nesting^76^. For example, Skibińska^71^ recorded only seven vespid species on a moist meadow on the Mazovian Lowland.

However, the above explanation of the lowest species richness and abundance of vespids in subcluster X_L_ cannot be satisfactory. X_L_ comprised samples from various habitats, of which only some were located on humid soil. Moreover, subcluster Y_S_ also contained some samples on humid soil and was the richest one in aculeates. The common feature for the diverse habitats of X_L_ was the presence of homogenous surrounding landscapes (Fig. 3a). In contrast, samples assigned to Y_S_ came from sites surrounded by significantly more diverse landscapes (Fig. 3a), which positively influenced the species richness and abundance of solitary and social vespids, including the sole dominant *P. nimpha* and the remaining social wasps (Fig. 4c,f,i,l,o). The presence of a more mosaic neighbourhood could increase the availability of often spatially separated resources necessary for wasps to complete their life cycle, such as nectar and pollen, prey, nest construction materials, refuges and overwintering sites^77–83^. Aculeates are central-place foragers, as they must build and provision the nest^11,83,84^; hence, they use various habitats within their flight range^85–87^. Similar effects of habitat mosaicity have been reported for moths^88^, butterflies^89,90^, plant-hoppers^91^, flea beetles^92^, bees^93,94^, trap nesting bees and wasps^82^, spider wasps^12^ and chrysidids^13^. For example, the species richness of sphecid wasps in moist meadows in Hungary was found to depend on the heterogeneity of the surrounding matrix rather than the conditions in the meadows sampled: a higher species richness was recorded in meadows located among dry sandy hills covered with flowering plants than in a meadow surrounded by neighbouring habitats with heavier soils^95^. Similarly, the species richness of bee and wasp assemblages was found to decrease as the mosaicity of agricultural areas was reduced: The attractiveness of agricultural areas drops if there are fewer field boundaries and roadsides which serve as refuges for bees and wasps^62,96,97^.

In the subcluster Y_D_, the reduced humidity of the soil resulted in a high number of social wasp species (Fig. 3g). This is connected with resource requirements^1,29,98–100^. The population dynamics of endogeic bees and wasps (ground-nesters) can be determined not only by the food resources present (pollen/nectar and insect prey) but also the availability of nesting places^12,13,100,101^. Endogeic species, particularly diggers, like the social wasps of the genus *Vespula*, prefer areas which are drier and more bare^3^, with a more friable soil^1,98^, instead of cold humid soils. This is in line with our present findings, in which the presence of endogeic vespids (i.e. social wasps and social wasps without dominant *Polistes nimpha*) was generally limited to sites with more exposed soil (Figs. 3b, 4h,n, and 5, Tables 3 and 4).

In contrast, hypergeic species, i.e. those nesting above the ground, are less limited in this respect as they may build nests in a variety of habitats, some of which may be unavailable to ground-nesters^38,98,102^. Almost all hypergeic wasps in this study were associated with subcluster X_W_ and Y_S_ containing samples from wooden buildings (X_W_) or samples from fallow land on semihumid and humid soil, often overgrown with vegetation (Y_S_). The solitary hypergeic species in our study (Figs. 3 and 4, Tables 3 and 4) may locate their nests in any empty space, such as holes made in dead wood by wood-boring insects or the empty stems of plants or twigs^19,29,38,84,103,104^; the social hypergeic species of the genus *Polistes* build aboveground exposed (open) paper nests, which are attached to substrates of various types (on various plants, including dwarf shrubs, twigs, thickets, or under the eaves of roofs and buildings)^1,38^.

Subcluster X_W_ contained samples collected almost exclusively on wooden buildings (Fig. 2, Table 2), and demonstrated the highest median number of solitary vespid species (Fig. 3e). Similarly, Wiśniowski^69^ found the highest species richness of solitary vespids (Eumenidae) in Ojców NP to be on wooden buildings and in ecotones. Similarly, high diversities of pompilid (all solitary) and chrysidid species (all cleptoparasites) were observed on wooden buildings in the Kampinos NP^12,13^, and in the Valle d’Aosta Region in the Italian Alps^105^. However, the solitary nature of aculeates inhabiting wooden buildings and their lower fecundity compared with social species^84^ results in a relatively low total abundance in such habitats (Fig. 3d).

The differences in the habitats exploited by vespids were reflected in the number of species exhibiting significant maximum IndVal in particular subclusters, i.e. their preference for respective environmental conditions. This number was highest in Y_S_ (five species) and intermediate in Y_D_ and X_W_ (four and three species, respectively) (Fig. 5, Table 3). No such species was observed in X_L_, comprising samples from sites with surrounding landscapes of the lowest mosaicity (Figs. 2 and 3a, Table 3). This indicates that the number of such species may serve as a bio-indicator of environmental quality for a given group of animals^12,13,106^, which is supported by the fact that the number of species with significant IndVals (Fig. 5, Table 3) corresponded to the species richness of vespids (Fig. 3c).

The species with significant maximum IndVals (group α), or those which were caught sporadically and present in samples assigned to only one subcluster (with A = 100% in group β) in Y_D_ were exclusively social, whereas those caught in X_W_ and Y_S_ were mostly solitary (Table 3).

The three species significantly associated with wooden buildings (X_W_) merit special attention. They nest obligatorily or facultatively above the ground (from nesting guilds *ii* and *iii*) and exhibit two different ecological amplitudes: *Polistes dominula* and *Ancistrocerus claripennis* Thomson, 1874 are eurytopic and are associated with open areas, while *Symmorphus murarius* (Linnaeus, 1758) is associated with woodland areas and forest edges. Of these, *A. claripennis* and *S. murarius* were not only indicators for X_W_ but were exclusively recorded in samples from this subcluster. They are solitary and xylicolous species, in that they build their nests in soft-core stems, hollow stems or soft pieces of wood. Their nests were found in doors and window frames, wooden walls of houses, and rotting beams and columns^38,107^. *Polistes dominula* is thermophilous and silviphobic; it occurs in various open habitats^28,34,67,68^ including ruderal habitats, fallow areas, loess and sandy grasslands, sand walls, weed vegetation, pastures and raspberry plantations^67,69,108^. It often inhabits anthropogenic biotopes^67,108–110^. Its occurrence is associated with human-made buildings^110^. It can attach nests to both vertical and horizontal substrates^1,34^, which makes nesting easier in various places. In Central Europe, it builds nests in sheltered places, e.g. window frames, shutters and doors^38^, under eaves, tiles, beams, eternit roofs, plates on locomotives and in gas boxes (Szczepko *unpublished*); hence, their preferred habitat in the KPN was (wooden) buildings. *Ancistrocerus claripennis* was recorded in ecotones, meadows, pastures, mixed forests, oak-hornbeam forests and xerotherms^34,38,111^ but most often on wooden or clay buildings and constructions^69,104,112^.

Moreover, subcluster X_W_ includes all the samples with four species: *Ancistrocerus antilope* (Panzer, 1798) and *A. gazella* (Panzer, 1798), which are eurytopic, and *Symmorphus allobrogus* (Saussure, 1855) and *S. connexus* (Curtis, 1826), which are associated with woodland areas and forest edges. All these species are recorded both in forests^34,60^ and in open habitats^38,66,111^, including those of anthropogenic origin: wooden constructions and old wooden or daubed buildings^69,104^.

All the above xylicolous species nest in burrows of beetle larvae in dry wood, which is more common in well-insulated places like the southern walls of wooden buildings^104,113^. Hence, the traps in our study were hung on the southern or western walls. The wooden buildings tested in the present study were 80 to 150 years old, not renewed and in most cases not impregnated^114^. The older buildings tended to display higher numbers of available borings and hollows of various diameters, which allowed the coexistence of species with different preferences^104^. In addition, the development of vespid assemblages may be promoted by the presence of gardens: flower nectar and honeydew serve as sources of food for adult wasps^115^, and gardens provide potential prey caught by vespid adults for the larvae, such as insect imagines for *Polistes*, and larvae of small butterflies and beetles for *Symmorphus* and *Ancistrocerus*^19,29,34,38,104^. These species associated with X_W_ are examples of “cultural species”^116^, which are common in anthropogenic habitats (“cultural” habitats)^117^.

The diversity of habitats of the studied post-agriculture area enables the occurrence of different mesofaunal predators which control populations of many insects, for instance caterpillars of microlepidoptera (e.g. *Eumenes coronatus, E. coarctatus, E. pedunculatus, Allodynerus delphinalis, Polistes nimpha* and *P. dominula*), larvae of beetles (e.g. *Symmorphus bifasciatus* (Linnaeus, 1761) and *S. murarius*) and weevils (e.g. *Microdynerus parvulus* (Herrich-Schaeffer, 1838) or dipterans (*Vespula germanica, V. rufa* (Linnaeus, 1758), *V. vulgaris*) (ex.^1,4,34,37,38,104^).

In summary, with reference to our hypotheses

1. The highest vespid abundance and richness were recorded in mosaic landscapes and in open habitats on semihydrogenic soil. They were also higher for social wasps, except *P. nimpha*, in open habitats with bare ground on dry soil (Fig. 4). The type of soil determines nesting resources for many vespids, especially those nesting in the ground (e.g. *Vespula germanica*, *V. rufa*). Landscape mosaicity widens the range of available resources to build and provision the nest within the flight range.
2. We managed to identify a single dominant habitat feature or a combination of 2–3 such features differentiating particular homogenous classes (SOM subclusters) of vespid samples: These were wooden buildings (for X_W_), open habitats on dry soil (for Y_D_), open habitats on (semi)humid soils in a mosaic landscape (for Y_S_) and low mosaicity of the surrounding area (for X_L_). The interesting observation was that X_L_ comprised samples from various habitats (fallow fields, an abandoned farm, forests and a meadow), and poorly diversified surrounding constituted their only common feature (Figs. 2 and 3).
3. No indicator species was found (based on IndVal) for X_L._ For the remaining homogenous classes (SOM subclusters) of vespid samples from three to five indicator species were identified. All indicator species were social in Y_D_, and mostly solitary in Y_S_. Concerning nesting preferences, the endogeic species were significantly associated only with subcluster Y_D_, the species nesting in or above the ground were associated with subclusters X_W_ and Y_D_, and the hypergeic species were associated with subclusters X_W_ and Y_S_ (Fig. 5, Tables 3 and 4). Therefore, the number of indicator vespid species identified by Indicator Species Analysis may serve as a bio-indicator of environmental quality for a given group of animals.

Our findings highlight the environmental importance of abandoned constructions of anthropogenic origin, such as wooden buildings, which clearly increase the structural diversity of the environment. Our results also show that not only the features of a sampled habitat but also the degree of heterogeneity of the surrounding matrix play an important role. As it has already been shown for pompilids and chrysidids^12,13,105^, among others, high landscape mosaicity promotes both increased species richness and abundance. Habitat heterogeneity positively influences faunal diversity, as each type of habitat widens the range of available, often spatially separated, resources, such as nectar and pollen, prey, nest construction materials, refuges and over-wintering sites^117^.

## Supplementary information

Dataset 1.
